# Association Between the Presence of Female-Specific Tumors and Aggressive Clinicopathological Features in Papillary Thyroid Cancer: A Retrospective Analysis of 9,822 Cases

**DOI:** 10.3389/fonc.2021.611471

**Published:** 2021-03-11

**Authors:** Jiao Zhang, Le Zhou, Gianlorenzo Dionigi, Daqi Zhang, Lina Zhao, Nan Liang, Gaofeng Xue, Hui Sun

**Affiliations:** ^1^ Division of Thyroid Surgery, China-Japan Union Hospital of Jilin University, Jilin Provincial Key Laboratory of Surgical Translational Medicine, Jilin Provincial Engineering Laboratory of Thyroid Disease Prevention and Control, Changchun, China; ^2^ Division for Endocrine and Minimally Invasive Surgery, Department of Human Pathology in Adulthood and Childhood “G. Barresi”, University Hospital G. Martino, University of Messina, Messina, Italy

**Keywords:** female, thyroid, benign tumors, papillary thyroid cancer, pathology, surgery

## Abstract

**Objective:**

To investigate the association between the presence of female-specific tumors and aggressive clinicopathological features in papillary thyroid cancer (PTC).

**Methods:**

This study retrospectively analyzed 9,822 female cases between June 2008 and December 2017. Odds ratios and corresponding 95% confidence intervals were calculated. Findings were stratified by age and body mass index (BMI) in different models.

**Results:**

1443/9822 (14.7%) patients with PTC had a female-specific tumor. Presence of a benign breast mass was an independent risk factor for a primary PTC lesion > 1 cm in diameter (adjusted OR = 1.446, 95% CI 1.136–1.840, *P* = 0.003), but a protective factor against extrathyroidal extension of PTC (adjusted OR = 0.650, 95%CI 0.500–0.845, *P* = 0.001). Presence of a benign uterine mass was an independent risk factor for multifocal PTC (adjusted OR = 1.305, 95%CI 1.113–1.531, *P* = 0.001). Analyses stratified by age and BMI revealed the presence of a benign breast mass was an independent risk factor for a primary PTC lesion > 1 cm in diameter in patients aged <36 years (adjusted OR = 1.711, 95% CI 1.063–2.754, *P* = 0.027), and a protective factor against extrathyroidal extension of PTC in patients aged ≥36 - <42 years (OR adjusted = 0.533, 95% CI 0.302–0.941, *P* = 0.030) or with a BMI ≥ 23.4 kg/m^2^ (BMI ≥ 23.4 to < 25.7 kg/m^2^, adjusted OR = 0.441, 95% CI 0.246–0.792, *P* = 0.006; BMI ≥25.7 kg/m^2^, adjusted OR = 0.558, 95% CI 0.315–0.998, *P*_2_ = 0.045). Presence of a benign uterine mass was an independent risk factor for multifocal PTC in patients aged ≥49 years (adjusted OR = 1.397, 95% CI 1.088–1.793, *P* = 0.009) or with a BMI <21.5 kg/m^2^ (OR adjusted = 1.745, 95% CI 1.214–2.509, *P* = 0.003).

**Conclusion:**

The presence of a benign breast mass was an independent risk factor for a primary PTC lesion > 1 cm in diameter and a protective factor against extrathyroidal extension of PTC, while the presence of a benign uterine mass was an independent risk factor for multifocal PTC. Data from this study may help surgeons propose more personalized treatment plans when encountering patients with PTC and female-specific benign tumors.

## Introduction

Evidence suggests that less women than men are affected by cancer. The National Cancer Institute estimates that one in three women and one in two men will be diagnosed with cancer during their lifetime, and women are more likely to survive cancer than men (1).

Despite this, the prevalence of thyroid cancer is higher in women than men (2–6), possibly due to the effects of endogenous female sex hormones, mood, stress, and genetic factors. Specifically, *in vitro* experiments show that estrogens promote the proliferation and invasion of thyroid cancer cell lines. Clinical data confirm a role for estrogen in the incidence of thyroid cancer (7–12), suggesting breast cancer and thyroid cancer share a common etiology (13). In one study, women with a history of breast cancer were 1.55 times more likely to develop thyroid cancer as a secondary malignancy compared to women with no history of breast cancer, while women with a history of thyroid cancer were 1.18 time more likely to develop breast cancer as a secondary malignancy compared to women with no history of thyroid cancer (14). These data imply that women with a history of breast or thyroid cancer should adhere to the appropriate guidelines for prevention of and screening for secondary malignancies during follow-up of their primary cancer.

The presence of female-specific tumors has also been associated with the incidence of thyroid lesions (15, 16). Benign breast (HR = 1.47, 95% CI 1.09–1.99) (17) and uterine (HR = 1.72, 95% CI 1.18–2.50) (13) masses may increase the risk of thyroid cancer. However, few reports have described the role of female-specific tumors in the progression of thyroid cancer.

The objective of this study was to investigate the association between the presence of female-specific tumors and aggressive clinicopathological features in papillary thyroid cancer (PTC). Understanding the molecular mechanisms responsible for sex-bias differences in thyroid cancer progression may improve the management of patients with thyroid cancer and inform the development of personalized therapeutic strategies.

## Methods

### Study Design and Data Sources

This retrospective analysis used data collected from an institutional prospective web-based registry platform. The study was conducted in compliance with the Declaration of Helsinki. The Health Care Ethics Committee of the China-Japan Union Hospital of Jilin University, Changchun, China, approved the protocol (NO. 2019040806). All patients had signed informed consent.

### Study Patients

**Figure 1:**
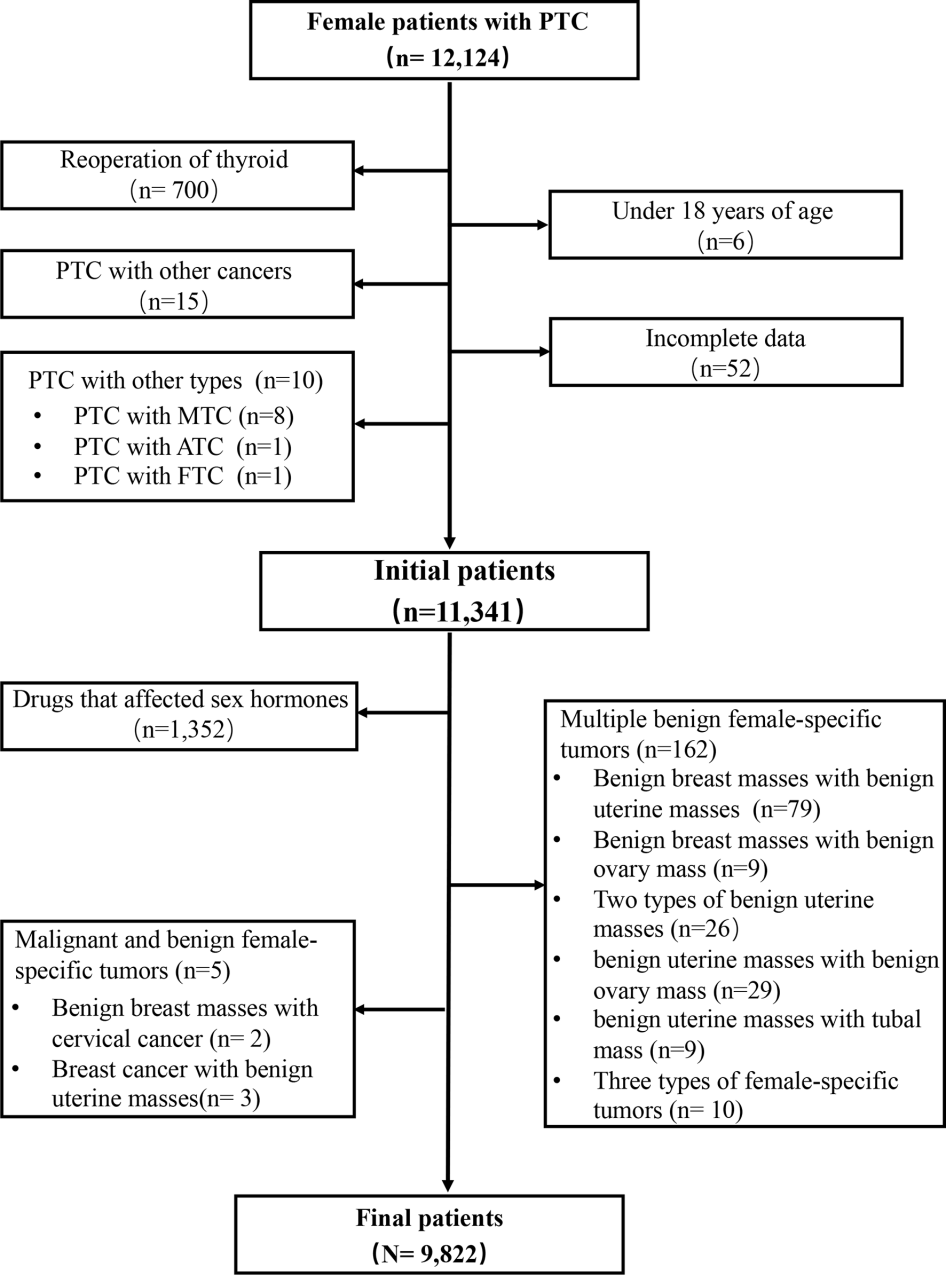
Flow chart of patient selection.

Patients with PTC (age ≥ 18 years) who were treated at the Division of Thyroid Surgery of the China-Japan Union Hospital of Jilin University (Changchun, China), a high-volume university-affiliated teaching hospital, between June 2008 and December 2017 were eligible for this study. Exclusion criteria were: 1) age < 18 years; 2) underwent reoperation; 3) multiple female-specific tumors; 4) history of other cancer or subtype of thyroid cancer; 5) long-term use of drugs that might affect the levels of sex hormones; 6) missing data (**Figure 1**).

Patients with PTC were classified into two groups according to the presence or absence of female-specific tumors, including benign breast masses, benign uterine masses, benign ovarian masses, breast cancer, or gynecological cancers. Benign breast masses included breast fibroadenoma, breast cysts, breast intraductal papilloma, and breast lipoma assigned a Breast Imaging Reporting and Data System (BI-RADS) 1–3 assessment on ultrasound and confirmed by long-term follow-up or biopsy (18). Benign uterine masses included uterine fibroids, uterine polyps, cervical cysts and uterine adenomyoma confirmed by ultrasound or pathology. Benign ovarian masses included ovarian cysts and mature teratoma confirmed by ultrasound or pathology. Breast cancers and gynecological cancers, including endometrial cancer, cervical cancer, and ovarian cancer were verified by postoperative paraffin pathology (**Table S1**).

PTC was diagnosed and characterized on postoperative paraffin pathology. Aggressive clinicopathological characteristics in PTC included a tumor diameter >1 cm, multifocal PTC, extrathyroidal extension of PTC, lymph node metastasis, T3-T4, clinical stage III or IV, and distant metastasis found on pathology or imaging in lung, bone, lung and bone combined, or other sites (19).

### Data Collection

Patients demographic and clinical characteristics were recorded, including height, weight, gender, age, previous medical history, thyroid function (thyroid stimulating hormone [TSH], tri-iodothyronine [FT3], thyroxine [FT4], thyroid peroxidase antibody [TPO-Ab], thyroglobulin [Tg] and thyroglobulin antibodies [Tg-Ab]), and clinicopathological characteristics of PTC (size of the primary lesion, number of lesions, extrathyroidal extension, number of lymph node metastases [including the central area and lateral neck area], distant metastasis, tumor, node and metastasis [TNM] and clinical staging). TNM and clinical staging were conducted according to the AJCC guidelines 8th edition (20).

### Statistical Analysis

Statistical analysis was performed using SPSS 22.0 for windows (SPSS, Chicago, IL, USA). Continuous data are reported as mean ± standard deviation and were compared using the t-test or analysis of variance. Categorical data are reported as percentages. and were compared with the chi-square test or Fisher’s exact test. Binary logistic regression was used to calculate odds ratios (OR) and 95% confidence intervals (CI). The independent variable was the coexistence of PTC and a female-specific tumor, and the dependent variables were the aggressive clinicopathological characteristics in PTC. Further binary logistic regression considered age or BMI as continuous variables in different models. ORs and corresponding 95% CIs were calculated to investigate the association between the presence of a benign breast mass and a primary PTC lesion > 1 cm in diameter, or extrathyroidal extension of PTC or the presence of a benign uterine mass and multifocal PTC. The subjects were divided into four categories based on quartiles of age and BMI in **Table 6** and **Table 7**. *P*<0.05 was considered statistically significant.

## Results

### Baseline Characteristics

A total of 12,124 female patients with PTC were identified from the institutional prospective web-based registry platform, of which 9,822 met the inclusion criteria (**Figure 1**). Included patients were all female, with a mean (SD) age of 42.91 ± 9.43 years, and a mean (SD) BMI of 23.71 ± 3.25 kg/m^2^. 85.3% (8379/9822) of patients with PTC did not have a female-specific tumor. 14.7% (1443/9822) of patients with PTC had a female-specific tumor. Of these, 4.3% (425/9822) of patients had a benign breast mass, 0.5% (47/9822) of patients had a breast cancer, 8.0% (790/9822) of patients had a benign uterine mass, 1.6% (156/9822) of patients had a benign ovarian mass, and 0.3% (25/9822) of patients had a gynecological cancer. Considering aggressive clinicopathological characteristics in PTC, 21.2% (2082/9822) of patients had a primary PTC lesion > 1 cm in diameter, 40.4% (3970/9822) of patients had multifocal PTC, 28.8% (2831/9822) of patients had extrathyroidal extension of PTC, 40.8% (4009/9822) of patients had lymph node metastasis, 4.6% (447/9822) were T3 or T4, and 0.2% (16/9822) of patients were clinical stage III or IV or had distant metastasis (**Table 1**).

**Table 1:** Baseline characteristics of 9,822 female of PTC.

Characteristic	Total (n = 9822)	Characteristic	Total (n = 9822)
	Mean ± SD or N (%)		Mean ± SD or N (%)
**Mean age**	42.91 ± 9.43	Extrathyroidal extension	28.8% (2831/9822)
**Height (cm)**	161.54 ± 4.70	**Tumor stage^1^**	
**Weight (kg)**	61.90 ± 9.04	T1a	77.1% (7575/9822)
**Mean BMI (kg/m^2^)**	23.71 ± 3.25	T1b	15.5% (1520/9822)
**Female-specific Tumors**	14.7% (1443/9822)	T2	2.8% (280/9822)
Benign Breast Masses	4.3% (425/9822)	T3a	0.1% (502/9822)
Benign Uterine Masses	8.0% (790/9822)	T3b	3.3% (323/9822)
Benign Ovary Masses	1.6% (156/9822)	T4a	1.2% (116/9822)
Breast Cancer	0.5% (47/9822)	High T stage	4.6% (447/9822)
Gynecological Cancers	0.3% (25/9822)	**Lymph node metastasis**	40.8% (4009/9822)
**Thyroid Function**		N0	59.2% (5813/9822)
TSH (mIU/L)	3.31 ± 3.68	N1a	25.6% (2520/9822)
FT3 (pmol/L)	4.47 ± 1.07	N1b	15.2% (1489/9822)
FT4 (pmol/L)	15.78 ± 6.76	**Clinical Stage^1^**	
Tg	19.71 ± 43.53	I	96.2% (9456/9822)
Tg-Ab	78.90 ± 306.29	II	3.6% (350/9822)
TPO-Ab	56.55 ± 120.99	III	0.1% (11/0922)
**Pathological features**		IVA	–
Mean tumor size (mm)	0.80 ± 0.60	IVB	0.1% (5/9822)
Tumor diameter >10 mm	21.2% (2082/9822)	**High clinical stage**	0.2% (16/9822)
Multifocality	40.4% (3970/9822)	**Distant metastasis**	0.2% (16/9822)

BMI, body mass index; TSH, thyroid stimulating hormone; FT3, free triiodothyronine; FT4, free thyroxine; TPO-Ab, thyroid peroxidase antibody; Tg-Ab, thyroglobulin antibody.

^1^Determined by 8th Edition of AJCC/UICC TNM Staging System.

### Association Between Female-Specific Tumors and Aggressive Clinicopathological Characteristics in PTC

Compared to patients with PTC without a female-specific tumor, more patients with PTC and a female-specific tumor had multifocal PTC (44.8%, vs. 39.7%, *P*<0.001) and less in lymph node metastasis (35.1% vs. 41.8%, *P*<0.01) (**Table 2**). There was correlation between PTC lesion > 1 cm and high BMI, younger age, high TSH and high Tg; between multifocality and high BMI, older age, high TPO-Ab, Tg-Ab; between ETE and high BMI, older age, high FT3, low TPO-Ab, high Tg, high Tg-Ab, low Tg/TSH; between lymph node metastasis and high BMI, younger age, high Tg, high Tg-Ab. When analyzing corresponding parameters, we would use related variables as covariates to adjust the OR value of aggressive clinicopathological features (**Table 3**).

**Table 2:** Aggressive clinicopathological features of PTC with or without female-specific tumors.

	Without female-specific tumors n (%)	With female-specific tumors n (%)	*P* value
PTC lesion > 1 cm	1769 (21.1%)	313(21.7%)	0.625
Multifocality	3324 (39.7%)	646(44.8%)	<0.001^**^
ETE	2439 (29.1%)	392(27.2%)	0.070
High T stage	383 (4.6%)	64(4.4%)	0.891
Lymph node metastasis	3502 (41.8%)	507(35.1%)	<0.001^**^
Distant metastasis	11 (0.1%)	5(0.3%)	0.073
High clinical stage	12 (0.1%)	4(0.3%)	0.199

ETE, extrathyroidal extension.

^**^ *P* < 0.01.

**Table 3:** The association between different variables and aggressive clinicopathological features of PTC.

	BMI	Age	TSH	FT3	FT4	TPO-Ab	Tg	Tg-Ab	Tg/TSH
PTC lesion ≤ 1 cm	23.62 ± 3.19	43.13 ± 9.03	3.23 ± 3.12	4.48 ± 1.11	15.75 ± 3.43	55.29 ± 119.83	16.51 ± 35.69	77.42 ± 299.742	28.37 ± 372.01
PTC lesion > 1 cm	24.06 ± 3.43	42.08 ± 10.75	3.61 ± 5.27	4.45 ± 0.90	15.93 ± 13.14	61.28 ± 125.17	31.72 ± 63.54	84.39 ± 329.489	39.84 ± 649.39
*P* value	<0.001^**^	<0.001^**^	0.002^**^	0.219	0.538	0.055	<0.001^**^	0.382	0.332
unifocality	23.49 ± 3.18	42.71 ± 9.66	3.35 ± 3.88	4.48 ± 1.21	15.71 ± 2.71	54.25 ± 123.05	19.22 ± 43.31	71.24 ± 281.15	33.50 ± 536.33
Multifocality	24.04 ± 3.32	43.20 ± 9.08	3.24 ± 3.37	4.48 ± 0.83	15.89 ± 10.12	59.94 ± 117.82	20.44 ± 43.84	90.21 ± 339.78	26.79 ± 257.63
*P* value	<0.001^**^	0.011^*^	0.166	0.996	0.205	0.023^*^	0.206	0.006^**^	0.495
non-ETE	23.61 ± 3.22	42.55 ± 9.34	3.30 ± 3.86	4.46 ± 1.15	15.79 ± 7.87	60.09 ± 128.53	17.41 ± 38.92	71.24 ± 281.87	36.07 ± 520.63
ETE	23.97 ± 3.31	43.80 ± 9.59	3.34 ± 3.20	4.52 ± 0.83	15.76 ± 2.40	47.79 ± 99.44	25.61 ± 53.07	98.44 ± 360.55	17.27 ± 106.01
*P* value	<0.001^**^	<0.001^**^	0.599	0.005^**^	0.856	<0.001^**^	<0.001^**^	0.001^**^	0.007^**^
Low T stage	23.69 ± 3.23	42.88 ± 9.40	3.31 ± 3.71	4.48 ± 1.09	15.79 ± 6.91	56.99 ± 121.64	19.04 ± 41.92	79.44 ± 306.43	31.24 ± 454.84
High T stage	24.08 ± 3.45	43.53 ± 10.09	3.32 ± 3.15	4.43 ± 0.68	15.60 ± 2.23	47.31 ± 106.18	34.11 ± 68.02	67.14 ± 303.43	20.96 ± 96.30
*P* value	0.013^*^	0.157	0.956	0.328	0.565	0.066	<0.001^**^	0.437	0.660
non-Lymph node metastasis	23.81 ± 3.14	44.71 ± 8.67	3.25 ± 2.94	4.47 ± 1.22	15.86 ± 8.33	57.61 ± 118.72	16.62 ± 35.36	68.97 ± 265.88	24.30 ± 259.09
Lymph node metastasis	23.56 ± 3.39	40.29 ± 9.88	3.38 ± 4.55	4.49 ± 0.80	15.68 ± 3.38	54.98 ± 124.26	24.33 ± 53.10	93.52 ± 357.14	40.44 ± 626.93
*P* value	<0.001^**^	<0.001^**^	0.078	0.361	0.205	0.297	<0.001^**^	<0.001^**^	0.154
non-Distant metastasis	23.71 ± 3.25	42.91 ± 9.42	3.31 ± 3.69	4.48 ± 1.07	15.78 ± 6.77	56.60 ± 121.08	19.68 ± 43.52	78.96 ± 306.51	30.74 ± 445.39
Distant metastasis	24.14 ± 3.97	44.69 ± 14.07	2.45 ± 1.53	4.68 ± 0.68	16.28 ± 2.11	23.09 ± 37.42	39.81 ± 44.15	37.16 ± 98.11	59.60 ± 156.73
*P* value	0.597	0.620	0.349	0.442	0.768	0.003^**^	0.084	0.610	0.808
Low clinical stage	23.71 ± 3.25	42.88 ± 9.41	3.31 ± 3.69	4.48 ± 1.07	15.79 ± 6.77	56.63 ± 121.08	19.65 ± 43.34	79.00 ± 306.540	30.71 ± 445.40
High clinical stage	24.47 ± 3.19	60.69 ± 6.24	2.84 ± 1.80	4.42 ± 0.65	15.79 ± 1.58	10.62 ± 5.88	58.06 ± 102.37	19.03 ± 22.23	72.12 ± 167.27
*P* value	0.351	<0.001^**^	0.615	0.838	0.995	<0.001^**^	0.168	0.449	0.719

ETE, extrathyroidal extension; BMI, body mass index; TSH, thyroid stimulating hormone; FT3, free triiodothyronine; FT4, free thyroxine; TPO-Ab, thyroid peroxidase antibody; Tg-Ab, thyroglobulin antibody.

^*^ *P* <0.05, ^**^ *P* <0.01.

On univariate analysis, compared to patients with PTC without a female-specific tumor, more patients with PTC and a benign breast mass had a primary PTC lesion > 1 cm in diameter (25.4% vs. 21.1%, *P*<0.05) but fewer patients with PTC and a benign breast mass had extrathyroidal extension of PTC (20.2% vs. 29.1%, *P*<0.001). Compared to patients with PTC without a female-specific tumor, more patients with PTC and a benign uterine mass had multifocal PTC (47.0% vs. 39.7%, *P*<0.001) or distant metastases (0.6% vs. 0, *P*<0.01), but fewer patients with PTC and a benign uterine mass had lymph node metastases (33.7% vs. 41.8%, *P*<0.001). The presence of a benign ovarian mass, breast cancer, or gynecological cancer was not associated with aggressive clinicopathological characteristics in PTC (**Table 4**). Binary logistic regression showed that the presence of a benign breast mass was an independent risk factor for a primary PTC lesion > 1 cm in diameter (adjusted OR = 1.446, 95% CI 1.136–1.840, *P* = 0.003), but a protective factor against extrathyroidal extension of PTC (adjusted OR = 0.650, 95%CI 0.500–0.845, *P* = 0.001). The presence of a benign uterine mass was an independent risk factor for multifocal PTC (adjusted OR = 1.305, 95%CI 1.113–1.531, *P* = 0.001) (**Table 5**).

**Table 4:** Univariate analysis of the association between female-specific tumors and aggressive clinicopathological features in PTC.

	Without Female-Specific Tumors (N=8379)	Benign Breast Masses(N=425)		Breast Cancer(N=47)		Benign Uterine Masses (N=790)		Benign Ovary Masses (N=156)		Gynecological Cancers (N=25)	
	n (%)	n (%)	*P*	n (%)	*P*	n (%)	*P*	n (%)	*P*	n (%)	*P*
PTC lesion > 1 cm	1769 (21.1%)	108 (25.4%)	0.039^*^	11 (23.4%)	0.720	149 (18.9%)	0.143	39 (25.0%)	0.236	6 (24.0%)	0.806
Multifocality	3324 (39.7%)	175 (41.2%)	0.542	22 (46.8%)	0.370	371 (47.0%)	<0.001^**^	67 (42.9%)	0.410	11 (44.0%)	0.685
ETE	2439 (29.1%)	86 (20.2%)	<0.001^**^	16 (34.0%)	0.519	233 (29.5%)	0.838	51 (32.7%)	0.329	6 (24.0%)	0.665
High T stage	383 (4.6%)	19 (4.5%)	1.000	3 (6.4%)	0.475	33 (4.2%)	0.720	8 (5.1%)	0.698	1 (4.0%)	1.000
Lymph node metastasis	3502 (41.8%)	158 (37.2%)	0.062	14 (29.8%)	0.104	266 (33.7%)	<0.001^**^	61 (39.1%)	0.513	8 (32.0%)	0.418
Distant metastasis	11 (0.1%)	0	1.000	0	1.000	5 (0.6%)	0.009^**^	0	1.000	0	1.000
High clinical stage	12 (0.1%)	0	1.000	1 (2.1%)	0.070	3 (0.4%)	0.133	0	1.000	0	1.000

ETE, extrathyroidal extension.

^*^ *P* <0.05, ^**^ *P* <0.01.

**Table 5:** Binary logistic regression analysis of the association between female-specific tumors and aggressive clinicopathological features in PTC.

		PTC lesion > 1 cm^a^	Multifocality^b^	ETE^c^	Lymph node metastasis^d^
Benign Breast Masses	Crude OR (95%CI)	1.273 (1.017–1.594)	1.065 (0.873–1.298)	0.618 (0.485–0.786)	0.824 (0.674–1.008)
	*P* value	0.035^*^	0.536	<0.001^**^	0.060
	Adjusted OR (95%CI)	1.446 (1.136–1.840)	1.016 (0.823–1.255)	0.650 (0.500–0.845)	0.875 (0.701–1.092)
	*P* value	0.003^**^	0.881	0.001^**^	0.236
Breast Cancer	Crude OR (95%CI)	1.142 (0.580–2.247)	1.338 (0.753–2.377)	1.257 (0.868–2.302)	0.591 (0.316–1.106)
	*P* value	0.701	0.320	0.459	0.100
	Adjusted OR (95%CI)	1.392 (0.650–2.983)	1.385 (0.710–2.700)	0.663 (0.300–1.467)	0.937 (0.455–1.932)
	*P* value	0.395	0.339	0.311	0.861
Benign Uterine Masses	Crude OR (95%CI)	0.869 (0.721–1.046)	1.347 (1.163–1.559)	1.019(0.868–1.196)	0.707 (0.606–0.824)
	*P* value	0.137	<0.001^**^	0.820	<0.001^**^
	Adjusted OR (95%CI)	0.965 (0.786–1.183)	1.305 (1.113–1.531)	0.984 (0.824–1.176)	0.877 (0.737–1.043)
	*P* value	0.729	0.001^**^	0.861	0.138
Benign Ovary Masses	Crude OR (95%CI)	1.246 (0.864–1.796)	1.145 (0.831–1.577)	1.183 (0.844–1.658)	0.894 (0.646–1.237)
	*P* value	0.240	0.407	0.330	0.449
	Adjusted OR (95%CI)	1.115 (0.718–1.729)	1.089 (0.760–1.561)	1.084 (0.726–1.619)	0.891 (0.610–1.303)
	*P* value	0.629	0.644	0.694	0.552
Gynecological Cancers	Crude OR (95%CI)	1.180 (0.471–2.959)	1.195 (0.542–2.635)	0.769 (0.307–1.928)	0.655 (0.283–1.520)
	*P* value	0.724	0.659	0.576	0.325
	Adjusted OR (95%CI)	1.366 (0.491–3.803)	1.431 (0.591–3.464)	0.589 (0.195–1.782)	1.477 (0.592–3.683)
	*P* value	0.550	0.427	0.349	0.403

OR, odds ratio; ETE, extrathyroidal extension.

^a^Binary logistic regression adjusted OR included, age, BMI, TSH and Tg as covariates.

b Binary logistic regression adjusted OR included, age, BMI, TPO-Ab, and Tg-Ab as covariates.

C Binary logistic regression adjusted OR included, age, BMI, TPO-Ab, FT3, Tg and Tg-Ab as covariates.

d Binary logistic regression adjusted OR included, age, BMI, Tg and Tg-Ab as covariates.

^*^ *P* <0.05, ^**^ *P* <0.01.

### Association Between Benign Breast Mass and Aggressive Clinicopathological Characteristics in PTC Stratified by Age and BMI

The presence of a benign breast mass was an independent risk factor for a primary PTC lesion > 1 cm in diameter in patients aged <36 years (adjusted OR = 1.711, 95% CI 1.063–2.754, *P* = 0.027). The presence of a benign breast mass was a protective factor against extrathyroidal extension of PTC in patients aged ≥36 - <42 years (OR adjusted = 0.533, 95% CI 0.302–0.941, *P* = 0.030).

The presence of a benign breast mass had no effect on the risk of developing a primary PTC lesion > 1 cm in diameter in patients with a BMI <21.5 - >25.7 kg/m^2^. The presence of a benign breast mass was a protective factor against extrathyroidal extension of PTC in patients with a BMI ≥ 23.4 kg/m^2^ (BMI ≥ 23.4 - < 25.7 kg/m^2^, adjusted OR = 0.441, 95% CI 0.246–0.792, *P* = 0.006; BMI ≥25.7 kg/m^2^, adjusted OR = 0.558, 95% CI 0.315–0.998, *P*_2_ = 0.045) (**Table 6**).

**Table 6:** Association between benign breast masses and aggressive clinicopathological features in PTC stratified by age and BMI.

	PTC lesion > 1 cm		ETE	
Model 1	OR^a^(95%CI)	*P* value	OR^b^(95%CI)	*P* value
Age quartiles (years)				
Age<36	1.711(1.063–2.754)	0.027^*^	0.726(0.402–1.311)	0.288
36≤Age<42	1.545(0.928–2.573)	0.095	0.533(0.302–0.941)	0.030^*^
42≤Age<49	1.286(0.789–2.096)	0.314	0.755(0.473–1.205)	0.238
Age≥49	1.490(0.923–2.405)	0.103	0.610(0.367–1.014)	0.056
Model 2				
BMI quartiles (kg/m^2^)				
BMI<21.5	1.581(0.979–2.552)	0.061	0.830(0.506–1.363)	0.462
21.5≤BMI<23.4	1.525(0.954–2.438)	0.078	0.770(0.475–1.246)	0.287
23.4≤BMI<25.7	1.215(0.733–2.014)	0.451	0.441(0.246–0.792)	0.006^**^
BMI≥25.7	1.559(0.964–2.521)	0.070	0.558(0.315–0.988)	0.045^*^

OR, odds ratio; BMI, body mass index; ETE, extrathyroidal extension.

a Binary logistic regression adjusted OR included, age, BMI, TSH and Tg as ccovariates.

b Binary logistic regression adjusted OR included, age, BMI, TPO-Ab, FT3, Tg and

Tg-Ab as covariates.

*P<0.05, **P<0.01.

**Figure 2:**
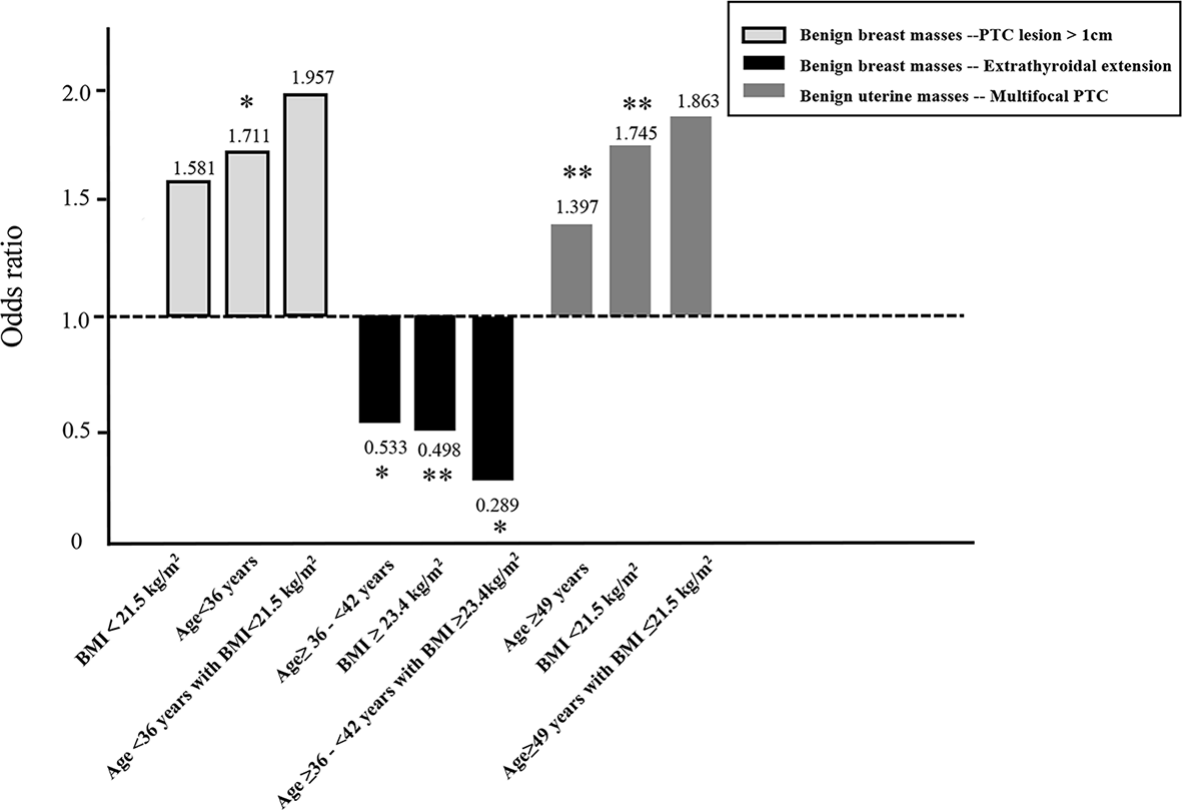
Influence of age and/or BMI on the association between the presence of a benign breast mass and a primary PTC lesion > 1cm in diameter or extrathyroidal extension of PTC, and the presence of a benign uterine mass and multifocal PTC. *P<0.05, **P<0.01.

The presence of a benign breast mass had no effect on the risk of developing a primary PTC lesion > 1 cm in diameter in patients aged <36 years with a BMI<21.5 kg/m^2^ (adjusted OR = 1.957, 95% CI 0.937–4.087, *P* = 0.074). The presence of a benign breast mass was a protective factor against extrathyroidal extension of PTC in patients aged ≥36 - <42 years with a BMI ≥23.4 kg/m^2^ (adjusted OR = 0.289, 95% CI 0.101–0.828, *P* = 0.021) (**Figure 2**).

In addition, age was a protective factor of PTC lesion >1 cm (adjusted OR _36≤age<42_ = 0.564, adjusted OR _42≤age<49_ = 0.513, adjusted OR _age≥49_ = 0.649). With age, the risk of extrathyroidal extension gradually increased (adjusted OR _36≤age<42_ = 1.189, adjusted OR _42≤age<49_ = 1.233, adjusted OR _age≥49_ = 1.420) **(** **Table S2** **)**.

### Association Between Benign Uterine Mass and Aggressive Clinicopathological Characteristics in PTC Stratified by Age and BMI

The presence of a benign uterine mass was an independent risk factor for multifocal PTC in patients aged ≥49 years (adjusted OR = 1.397, 95% CI 1.088–1.793, *P* = 0.009) or a BMI <21.5 kg/m^2^ (OR adjusted = 1.745, 95% CI 1.214–2.509, *P* = 0.003) (**Table 7**).

**Table 7:** Association between benign uterine masses and aggressive clinicopathological features in PTC stratified by age and BMI.

	Multifocal PTC	
Model 1	OR^a^(95%CI)	*P* value
Age quartiles (years)		
Age<36	0.989(0.495–1.987)	0.975
36≤Age<42	1.112(0.755–1.636)	0.592
42≤Age<49	1.289(0.987–1.683)	0.062
Age≥49	1.397(1.088–1.793)	0.009^**^
Model 2		
BMI quartiles (kg/m^2^)		
BMI<21.5	1.745(1.214–2.509)	0.003^**^
21.5≤BMI<23.4	1.266(0.883–1.813)	0.199
23.4≤BMI<25.7	1.331(0.999–1.773)	0.051
BMI≥25.7	1.095(0.819–1.463)	0.542

OR, odds ratio; BMI, body mass index.

a Binary logistic regression adjusted OR included, age, BMI, TPO-Ab and Tg-Ab as covariates.

**P < 0.01.

The presence of a benign uterine mass had no effect on the risk of developing multifocal PTC in patients aged ≥49 years with a BMI ≤21.5 kg/m^2^ (adjusted OR = 1.863, 95% CI = 0.977–3.554, *P* = 0.059) (**Figure 2**).

## Discussion

This retrospective analysis of 9,822 female patients investigated the association between the presence of female-specific tumors and aggressive clinicopathological features in PTC. A benign breast mass was an independent risk factor for a primary PTC lesion > 1 cm in diameter and a protective factor against extrathyroidal extension of PTC, while the presence of a benign uterine mass was an independent risk factor for multifocal PTC. Findings revealed that the presence of benign breast or uterine masses could influence invasive growth of PTC. Patients were stratified by age and BMI. In young female (age <36 years) patients, the presence of a benign breast mass was an independent risk factor for a primary PTC lesion > 1 cm in diameter. Among young and middle-aged overweight/obese women (age 36–42 years; BMI ≥ 23.4 kg/m^2^), the presence of a benign breast mass was a protective factor against extrathyroidal extension of PTC. In middle-aged (>49 years) or lean (BMI<21.5 kg/m^2^) patients, the presence of a benign uterine mass was an independent risk factor for multifocal PTC. This study demonstrated that the presence of female-specific benign tumors influences aggressive clinicopathological features in PTC. These data have implications for PTC surveillance and treatment.

Benign breast masses are commonly found in women. The prevalence of benign breast masses in China is estimated at 18.6% (16). In this study, 4.3% of patients had PTC and a benign breast mass. Previous studies have shown that postmenopausal women with a benign breast mass have a higher risk of developing thyroid cancer than women without a benign breast mass (HR = 1.38, 95% CI 1.10–1.73) (21), or the presence of a benign breast mass increased the risk of thyroid cancer by 47% (HR = 1.47, 95%CI 1.09–1.99) (17, 20). However, data describing the influence of a benign breast mass on the progression of thyroid cancer are scarce.

The present study revealed that the presence of a benign breast mass was a risk factor for a primary PTC lesion > 1 cm in diameter in women aged < 36 years(adjusted OR = 1.711, *P* = 0.027), but not in 36≤Age<42 (adjusted OR = 1.545, *P* = 0.095), 42≤Age<49 (adjusted OR = 1.286, *P* = 0.314), Age≥49 (adjusted OR = 1.490, *P* = 0.103).We suppose that the mechanism underlying the co-occurrence of a benign breast mass and thyroid cancer may involve estrogen. Estrogen plays an important role in the incidence and progression of benign breast masses (19, 22), and estrogen receptor expression is a common occurrence in thyroid tumor tissues. Vannucchi et al. proved that ERα and PR expression was found in 66.5% and 75.8% of patients respectively and was significantly correlated with larger tumor size and with a non-incidental diagnosis. A trend toward a higher prevalence of local metastases was observed in ER- and PR-expressing tumors (23). Similar to our study, the author concluded that although no impact on outcome was found, the evaluation of ERα and PR receptor expression could add insights into the biological behavior of tumors and could modify the follow-up, particularly in fertile women affected with persistent disease. And *in vitro* studies show that estrogens promote the proliferation of thyroid cancer cells (24–27). Ovarian reserve begins to decline at 35 years of age, the number of ovarian follicles and the quality of oocytes decreases, and estrogen secretion is reduced (28–30).

Besides, one study hypothesized that differences between young and older patients with PTC had a biological/genetic basis (31). However, they didn’t find a difference in the frequency or type of somatic mutations between young and older patients, six genes (extracellular matrix protein 1 [ECM1], v‐erb‐2 erythroblastic leukemia viral oncogene homolog 2 [ERBB2], urinary plasminogen activator [UPA], 6‐phosphofructo‐2‐kinase/fructose‐2,6‐biphosphatase 2 [PFKFB2], meis homeobox 2 [MEIS2], and carbonic anhydrase II [CA2]) had significant expression between the young and older patients. In another study, age was a protective factor of the primary PTC lesion > 1 cm (32). In present study, the presence of female-specific tumors was the risk factor for PTC lesion > 1 cm. With increasing age, this effect might be offset.

Interestingly, the presence of a benign breast mass was a protective factor against extrathyroidal extension of PTC. According to the data, age was a risk factor for extrathyroidal extension. With age, the risk of extrathyroidal extension gradually increased (adjusted OR _36≤age<42_ = 1.189, adjusted OR _42≤age<49_ = 1.233, adjusted OR _age≥49_ = 1.420). The protective effect of benign breast tumors might counteract the risk of extrathyroidal extension conducted related to age. It is clear that this could only be a hypothesis, it must be supported by clinical and experiemntal studies. And the effect that is unlikely to be explained by a mechanism involving estrogen (12, 33–35). but might be mediated by the tumor microenvironment. We speculate that in benign breast conditions, the immune system is challenged, and inflammation in the mammary gland may influence the thyroid gland to initiate a ‘self-protection’ mechanism, which prevents thyroid cancer invading through the capsule. Consequently, women with PTC and benign breast tumors may have larger diameter primary lesions but no extrathyroidal extension.

Stratifying patients by age and BMI demonstrated that the protective effect of benign breast masses on extrathyroidal extension of PTC was significant in overweight/obese women aged 36 to 42 years. Evidence suggests anti-Müllerian hormone is obviously decreased in women in their upper reproductive years, which is indicative of ovarian dysfunction. We speculate that fluctuations in anti-Müllerian hormone may mediate extrathyroidal extension of PTC (36).

Benign uterine masses are commonly found in women, with an estimated prevalence of 4.5% to 68.6% (37). The prevalence of benign uterine masses is age dependent, reaching > 70% in women aged > 50 years (38–40). One previous study showed that the presence of a benign uterine mass was associated with an increased risk of thyroid cancer (HR = 1.72, 95% CI 1.18–2.50) (13); however, data describing the influence of a benign uterine mass on the progression of thyroid cancer is scarce.

The present study revealed that the presence of a benign breast mass was a risk factor for a primary PTC lesion > 1 cm in diameter in women aged < 36 years. The multifocality of PTC has been explained at the genetic level in different ways, being two hypothesis the more relevant to date: a) different foci composed by different clones with different genetic pattern; b) intrathyroidal metastatization. Some studies suggest discordant heterogeneous BRAF mutation patterns found in approximately 40% of the multifocal PTCs in adults (41). Multifocal PTCs had higher expression of mRNAs in Wnt- and pluripotency‐related pathways when BRAF mutation was present (42). It is still primitive to give conclusions about the mechanisms, but these appear to be the most credible to explain the difference between multifocal and unifocal PTCs.

In addition, it was proved that programmed death-ligand 1 (PD-L1) expression was elevated in multifocal PTC tumors (43). The expression of programmed death-1 (PD-1) and its ligand PD-L1 is upregulated in uterine disease and promoted by 17beta-estradiol (44). Therefore, we speculated the presence of a benign uterine mass was an independent risk factor for multifocal PTC.

We found that there was correlation between PTC lesion > 1 cm and high BMI, younger age, high TSH and high Tg; between multifocality and high BMI, older age, high TPO-Ab, Tg-Ab; between ETE and high BMI, older age, high FT3, low TPO-Ab, high Tg, high Tg-Ab, low Tg/TSH; between lymph node metastasis and high BMI, younger age, high Tg, high Tg-Ab. When analyzing corresponding parameters, we would use related variables as covariates to adjust the OR value of aggressive clinicopathological features. Besides, Preoperative Tg levels may have an extremely limited value. They could be influenced by several factors not related to thyroid cancer, for example gland size (45). Thus, we analyzed Tg/TSH levels and found that lower Tg/TSH was a risk factor of ETE. These findings were similar to other studies (32, 45–49).

This research had several strengths. First, the study revealed the association between the presence of female-specific tumors and aggressive clinicopathological features in PTC. Second, it was a relatively large single-center retrospective analysis of the relationship between age, BMI, female-specific tumors and PTC. Last, all cases of thyroid cancer were confirmed by medical records, avoiding potential differential selection and recall bias.

This study was assoictaed with several limitations. First, it addresses an evidence gap — is there an etiologic link between benign tumors and aggressive clinicopathological features in PTC. The study used a large sample size to fill this evidnce gap, but the precise mechanism underlying the co-occurrence of a benign breast/uterine mass and thyroid cancer has yet to be clearly defined. Second, as the study had a retrospective design without follow-up data, the impact on prognosis is unknown. Third, age of diagnosis of female-specific tumors and PTC were not confirmed; therefore, cause and effect cannot be established. Last, women with small benign tumors that were undetected may have been included in the analysis. Large-scale randomized controlled studies are warranted to provide further clarity to the influence of female-specific tumors on aggressive clinicopathological features in PTC.

In conclusion, this study retrospectively analyzed data collected from patients with PTC and a female-specific tumor in a single institutional database to reveal the association between the presence of a female-specific tumor and PTC proliferation and invasion. Findings showed the presence of a benign breast mass was an independent risk factor for a primary PTC lesion > 1 cm in diameter and a protective factor against extrathyroidal extension of PTC, while the presence of a benign uterine mass was an independent risk factor for multifocal PTC. The underlying mechanisms may be mediated by estrogen. Data from this study may help surgeons propose more personalized treatment plans when encountering patients with PTC and female-specific tumors.

## Data Availability Statement

The data sets generated and/or analyzed during the current study are not publicly available to protect patient privacy but are available from the corresponding author on reasonable request. Requests to access the data sets should be directed to HS,s_h@jlu.edu.cn.

## Ethics Statement

This study was approved by the Health Care Ethics Committee of the China-Japan Union Hospital of Jilin University (no. 2019040806). The patients/participants provided their written informed consent to participate in this study.

## Author Contributions

Conception/design: HS and JZ. Collection and assembly of data: JZ, DZ, LNZ, NL, and GX. Data analysis and interpretation: JZ and LZ. Manuscript writing: JZ and GD. Final approval of manuscript: JZ, LZ, GD, DZ, LNZ, NL, GX, and HS. All authors contributed to the article and approved the submitted version.

## Conflict of Interest

The authors declare that the research was conducted in the absence of any commercial or financial relationships that could be construed as a potential conflict of interest.
