# Supplementary material for: Association Between the Presence of Female-Specific Tumors and Aggressive Clinicopathological Features in Papillary Thyroid Cancer: A Retrospective Analysis of 9,822 Cases
Source: Front Oncol. 2021 Mar 11;11:611471. doi: 10.3389/fonc.2021.611471 (PMC8006326; doi:10.3389/fonc.2021.611471)
Supplement: Supplementary file 1 [file DataSheet_1.docx]

**Supplemental Table S1: Definition of female-specific tumors**

| **Definition** | **Total (n，%)** |
| --- | --- |
| Benign Breast Masses | 425 (29.5%) |
| Breast Fibroadenoma | 390 (91.8%) |
| Breast Cysts | 13 (3.1%) |
| Breast Intraductal Papilloma | 21 (4.9%) |
| Breast Lipomas | 1 (0.2%) |
| Breast Cancer | 47 (3.3%) |
| Benign Uterine Masses | 790 (54.7%) |
| Uterine Fibroids | 707 (89.5%) |
| Uterine Polyps | 27 (3.4%) |
| Cervical Cysts | 55 (7.0%) |
| Uterine Adenomyoma | 1 (0.1%) |
| Benign Ovary Masses | 156 (10.8%) |
| Ovarian Cysts | 131 (84.0%) |
| Mature Teratoma | 25 (16.0%) |
| Gynecological Cancers | 25 (1.7%) |
| Endometrial Cancer | 4 (16%) |
| Cervical Cancer | 8 (32%) |

**Supplemental Table S2:** **Association between age and aggressive clinicopathological features in PTC**

|  | Tumor lesion > 1cm |  | Extrathyroidal extension | |
| --- | --- | --- | --- | --- |
| Age quartiles | OR^a^（95%CI) | *P* value | OR^b^（95%CI) | *P* value |
| Age＜36 | Reference |  | Reference |  |
| 36≤Age＜42 | 0.564 (0.483-0.660) | ＜0.001^**^ | 1.189 (1.025-1.379) | 0.022^*^ |
| 42≤Age＜49 | 0.513 (0.441-0.597) | ＜0.001^**^ | 1.233 (1.069-1.422) | 0.004^**^ |
| Age≥49 | 0.649 (0.561-0.749) | ＜0.001^**^ | 1.420 (1.235-1.634) | ＜0.001^**^ |

**Abbreviations:** OR=odds ratio

^a^ Binary logistic regression adjusted OR included, age, BMI, TSH and Tg as covariates.

^b^ Binary logistic regression adjusted OR included, age, BMI, TPO-Ab, FT3, Tg and Tg-Ab

as covariates.
